# Depression Affects the Scores of All Facets of the WHOQOL-BREF and May Mediate the Effects of Physical Disability among Community-Dwelling Older Adults

**DOI:** 10.1371/journal.pone.0128356

**Published:** 2015-05-26

**Authors:** Yu-Chen Chang, Grace Yao, Susan C. Hu, Jung-Der Wang

**Affiliations:** 1 Department of Community Health, Chia-Yi Christian Hospital, Chia-Yi, Taiwan; 2 Division of Geriatrics, Chia-Yi Christian Hospital, Chia-Yi, Taiwan; 3 Department of Psychology, National Taiwan University, Taipei, Taiwan; 4 Department of Public Health, College of Medicine, National Cheng Kung University, Tainan, Taiwan; 5 Departments of Internal Medicine and Occuptional and Environmental Medicine, National Cheng Kung University, Tainan, Taiwan; Yeshiva University Albert Einstein College of Medicine, UNITED STATES

## Abstract

**Background:**

Geriatric depression is associated with the overall quality of life (QOL). However, how depressive symptoms affect the different domains and facets of QOL in older adults, and whether depressive symptoms mediate the relationship between physical disability and QOL in older adults are unclear.

**Methods:**

A total of 490 ambulatory community-dwelling older adults aged 65 years or above were interviewed using the brief version of the World Health Organisation Quality of Life instrument (WHOQOL-BREF), the Modified Barthel Index (MBI), the 15-item Geriatric Depression Scale (GDS-15), and the Mini-Mental State Examination (MMSE). Sequential models for multiple linear regressions were analysed to determine if the MBI, GDS-15 and MMSE scores predict the WHOQOL-BREF scores. The potential mediation effects of depression (as determined by the GDS-15) on the relationship between MBI and WHOQOL-BREF were also analysed.

**Results:**

The GDS-15 score was predictive of the scores of the four domains and all 26 facets of the WHOQOL-BREF. The significant predictive effects of the MBI score on 15 of the 26 facets of the WHOQOL-BREF were reduced to three after the adjustment for the GDS-15 score. Depression (as assessed by the GDS-15) is a mediator of the relationship between MBI and the physical, psychological and environmental domains of the WHOQOL-BREF.

**Conclusions:**

Depression (assessed by the GDS-15) may affect the scores of every domain and all facets of the WHOQOL-BREF in the elderly. Furthermore, it may mediate the relationship between the MBI and on QOL scores. We recommend taking depressive symptoms into consideration when measuring community-dwelling older adults’ QOL and providing active ageing programs.

## Introduction

Geriatric depression is an important public health issue. The average prevalence of depressive symptoms has been reported to be 13.5% among people who are 55 years or older [1]. It has been estimated to be more than 20% in the Chinese population aged 65 years and over in Taiwan [2].

Depression has been suggested as a strong predictor of the total score of quality of life (QOL) in older adults [3–5], and the QOL of people with major depression has been shown to improve after effective anti-depressant treatment [4,6]. In fact, an improvement of QOL has been considered a treatment goal of depression in older people [7]. In addition to affecting the overall condition of QOL, depression influences different facets of the different domains of QOL. A previous study showed that depression affects every facet of the brief version of the World Health Organisation Quality Of Life (WHOQOL-BREF) measurement in healthy workers[8]. However, it is less clear how depressive symptoms affect the QOL domains and facets in older people who are generally retired from work.

In addition, the limitations of mobility or activities of daily living (ADL) in older adults have been reported to decrease QOL [3,9,10]. In fact, impairments of ADL may have a greater effect than do chronic diseases [9]. Thus, it is important to include measurements of ADL in the prediction of the QOL score. However, many studies have neglected to include these measurements. Moreover, people with ADL difficulties but without anxiety or depressive symptoms have been found to have a higher QOL score than those with similar psychiatric morbidity [11]. Furthermore, physical disabilities may increase the risk of depressive symptoms in older adults [3,12]. These findings may suggest a possible mediation effect of depression on the relationship between ADL and QOL among older adults.

The primary objective of the current study, therefore, is to analyse the effects of depressive symptoms on QOL scores. Furthermore, this study aims to explore the possible mediating effect of depressive symptoms on the relationship between ADL and QOL scores in community-dwelling older adults.

## Methods

### Study design

This study was approved by the Institutional Ethics Committee of Chia-Yi Christian Hospital before commencement (registry number 101064). After providing informed consent, all participants were invited to complete questionnaires and were evaluated with the modified Barthel index (MBI) and Mini-Mental Sate Examination (MMSE) to measure their physical and mental conditions, respectively. We recruited community-dwelling older adults without severe functional dependence (MBI<60) for this study. The participants’ demographic characteristics and medical histories, including birth date, gender, education, marital status, household cohabitants, and cognitive function, ADL, and depression scores, were collected. We applied three sequential models to explore the relationship between the WHOQOL-BREF and the determinants.

### Participants

The participants were recruited from community centres located in both the northern and southern parts of Taiwan. These community centres provide active ageing programs, which are financed by the government. Invitation letters were mailed to each household, and the eligibility criteria included those aged 65 years and above who were able to walk for more than 100 metres without resting and also willing to answer the questionnaire. Eligible participants were enrolled after providing the written informed consent. People with severe cognitive impairment (MMSE < 18) [13] or severe dependency (MBI < 60) [14] were excluded.

### Measurements

#### Demographic and clinical variables

We collected the following socio-demographic data on the participants: age, which was classified into three groups (65–74 years, 75–84 years, and 85 years and older); education, which was classified as no formal education, elementary school, junior high school and above; marital status, which was classified as married and not married; and household cohabitants, which was classified as living alone or with family.

#### Quality of life (QOL)

The World Health Organisation (WHO) initiated a cross-cultural project to develop the standard 100-item World Health Organization Quality of Life instrument (WHOQOL-100) in 1991. Then, the WHOQOL research group simplified the WHOQOL-100 into a brief version called the WHOQOL-BREF [15]. This measure was culturally adapted into a Taiwan Chinese version [16,17], which is widely used in Taiwan [18,19]. The WHOQOL-BREF includes 2 general items and 24 items that represent 24 specific facets of the WHOQOL-100. The 24 items are categorised into four domains: physical, psychological, social relationships and environmental. Each facet is scored from 1 to 5 points, with a higher score indicating a better QOL. Each domain score ranges from 4 to 20 and is calculated by multiplying the average score of all facets of the respective domain by 4.

#### Depressive symptoms

The 15-item Geriatric Depression Scale (GDS-15) was used to measure depressive symptoms in the subjects selected for this study. This version was first described by Sheikh and Yesavage in 1986, and has been widely used around the world [20]. The Chinese version has been validated as having good sensitivity and specificity [21]. The sensitivity and specificity, with a cut-off point of 5 (score>5), are 71.8% and 78.2%, respectively [22].

#### Activities of daily living (ADL)

The Modified Barthel Index (MBI) was used to measure ADL performance for physical function impairment. The Barthel Index to measure ADL was first developed by Barthel [23]. It contains ten variables, and scores range from 1 to 20. Granger et al. [24] modified the scale in 1979 to include 0–10 points for each variable, with a total score of 100. This index was further improved and modified in 1989 [25]. Scores of 0–20 indicate total dependence; 21–60: severe dependence; 61–90: moderate dependence and 91–99: slight dependence [14,26]. People with an MBI<60 were excluded from the current study.

#### Cognitive function

We applied the Mini-Mental State Examination (MMSE) to measure cognitive function. The MMSE was developed by Folstein [27] and is commonly used to examine the cognitive function of older adults. The MMSE contains 30 items, and each item is assigned a binary score (1 for a correct answer and 0 otherwise). The highest possible score is 30. A recent study showed the sensitivity and specificity of the MMSE for Alzheimer’s disease, with a cut-off point of equal to or less than 26, were 0.79 and 0.90, respectively, in highly educated people [28]. Many studies have used a convenient cut-off point of 18 to identify severe cognitive impairment [13,29,30]. We excluded people with an MMSE score < 18 from this study and included the MMSE score as a controlled variable in this analysis.

#### Multi-morbidity

Five chronic conditions, including diabetes, hypertension, heart disease, stroke and cancer, were measured. All of these conditions were analysed as independent variable in the current study.

To obtain the above information, trained interviewers administered a standardised face-to-face assessment to all the participants. The interviews were conducted at the community centres. All of the interviewers had at least 12 years of education.

### Statistical analysis

Multiple linear regression models were constructed to analyse the sequential relationships of different levels of covariates within the four domains and each facet of the WHOQOL-BREF. During the construction of the models, we included gender, age, education, marital status, household cohabitants, multi-morbidity (including diabetes, hypertension, heart disease, stroke and cancer), the MMSE score, the GDS-15 score and the MBI as covariates. Model 1 included all of the covariates listed above except the GDS-15. Model 2 included all of the covariates except the MBI, and Model 3 included all covariates. Sequential models of the multiple linear regressions were used to analyse the relationship between the MBI and/or the GDS-15 score and the 26 items of the WHOQOL-BREF with adjustments for the covariates, including demographic and medical factors.

**Fig 1:**
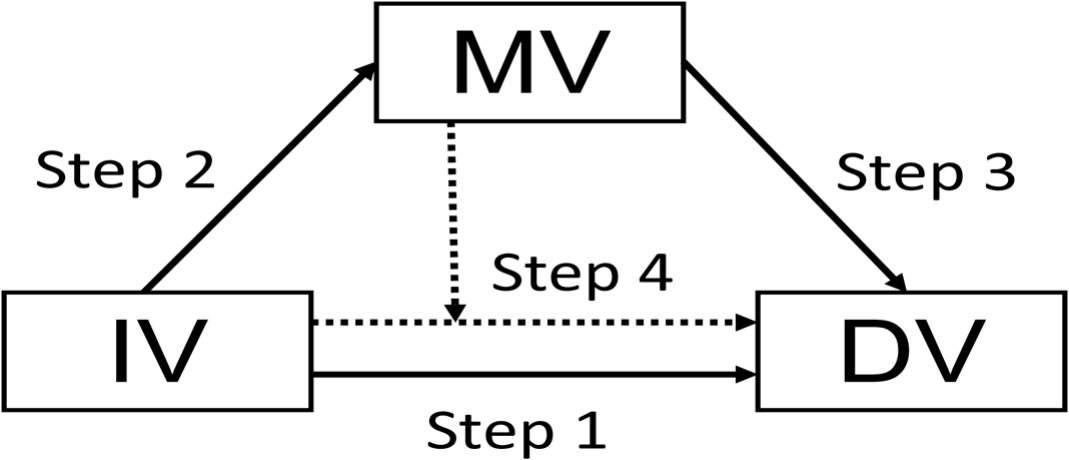
The scheme of testing procedure of mediating effects. IV: independent variable; MV: mediating variable; DV: dependent variable. Step 1: to test if IV predicts DV without including MV as a covariate; Step 2: to test if IV predicts MV; Step 3: to test if MV predicts DV; Step 4: to test if IV predicts DV with MV as a covariate.

The mediation effects of depression were tested according to the procedures of Baron and Kenny [31], which are shown in Fig 1. According to their research, the following four criteria are required to conclude mediation: Step 1: the independent variable (IV) significantly predicts the dependent variable (DV); Step 2: the IV significantly predicts the mediating variable (MV); Step 3: the MV significantly predicts the DV; Step 4: after adjusting for the MV, the association between the IV and the DV is reduced (partial mediation) or is no longer significant (complete or perfect mediation). Multiple regressions controlling for age, gender, education, marriage, household cohabitants, MMSE score, diabetes, hypertension, heart disease, stroke and cancer were conducted. Any variable that showed significant associations with any domain was treated as an IV and tested with the four criteria described above, and the GDS-15 was assumed as the MV. A Sobel test was performed when any set of IVs and MVs within the respective domain score met the four criteria for mediation effects [32].

The tasks were conducted using the SAS statistical package (version 9.2 for windows; SAS Institute, Inc., Cary, NC). The significance level was set at α< 0.05.

## Results

A total of 516 participants completed the questionnaire. Twenty-six participants did not fulfil the inclusion criteria, and 490 (95%) were included in the final analysis. The basic characteristics of the subjects are summarised in the first column of Table 1. Approximately two-thirds of the subjects were female; more than half were older than 75; approximately one-third had no formal education; half were married; and one-fourth were living alone.

**Table 1:** Basic demographic and medical characteristics of participants and coefficients and standard errors (S.E.) of major determinants for WHOQOL-BREF domain scores under multiple linear regression models.

	^┼^N or mean±SD	^┿^Phy Dom	^┿^Psy Dom	^┿^Soc Dom	^┿^Env Dom
		Est.(S.E.)	Est.(S.E.)	Est.(S.E.)	Est.(S.E.)
Gender					
Female/male	317/171	0.19(0.26)	0.03(0.26)	0.09(0.26)	0.12(0.24)
Age					
75-84/≦74y	212/195	-0.44(0.26)	-0.21(0.26)	-0.18(0.26)	-0.01(0.24)
≧85/≦74y	69/195	0.61(0.38)	0.38(0.37)	0.85(0.37)*	0.64(0.34)
Education					
Elementary/no education	167/148	0.19(0.30)	-0.08(0.29)	0.11(0.29)	0.01(0.27)
>Junior high/no education	148/148	0.29(0.31)	-0.08(0.31)	-0.65(0.31)	-0.00(0.28)
Marriage					
Not married/married	239/243	-0.21(0.25)	-0.17(0.24)	-0.26(0.24)	-0.21(0.22)
Cohabitant					
With family/alone	363/114	0.37(0.30)	0.30(0.30)	-0.02(0.30)	0.54(0.28)
MMSE	26±3.4	0.06(0.04)	0.00(0.04)	0.06(0.04)	0.03(0.03)
Diabetes	87	-0.02(0.31)	-0.14(0.30)	0.02(0.30)	0.37(0.28)
Hypertension	226	-0.05(0.25)	0.24(0.24)	0.46(0.24)	0.15(0.22)
Heart disease	110	-0.35(0.28)	-0.53(0.28)	0.06(0.28)	-0.28(0.26)
Stroke	21	0.00(0.60)	0.27(0.59)	0.68(0.59)	0.93(0.55)
Cancer	20	-0.05(0.58)	-0.20(0.57)	0.49(0.57)	0.45(0.52)
MBI	96.2±8.1	0.03(0.02)	0.02(0.02)	0.00(0.02)	0.01(0.01)
GDS	3.5±3.2	-0.23(0.04) ***	-0.28(0.04) ***	-0.18(0.04) ***	-0.21(0.04) ***

*p<0.05,

**p<0.01,

***p<0.001

^┼^Basic demographic and medical characteristics of participants

^┿^regression coefficients and standard errors (S.E.) of major determinants for WHOQOL-BREF domain scores under multiple linear regression models.

WHOQOL = World Health Organization Quality of Life Scale-Brief Version; MMSE = Mini-Mental State Examination; MBI = Modified Barthel Index; GDS = Geriatric Depression Scale; Phy Dom = Physical domain; Psy Dom = Psychological domain; Soc Dom = Social relationship domain; Env Dom = Environmental domain

The regression coefficients of multiple linear regression models for the scores of different domains of the WHOQOL are also shown in Table 1. The GDS-15 score was significantly predictive of all four domains of the WHOQOL-BREF. Being older than 85 was associated with a better quality of social relationships. There was no significant association between individual items of the multi-morbidity assessment and the scores of the four domains.

A further analysis of the association between the MBI and GDS-15 on each facet of the WHOQOL-BREF in three sequential models is summarised in Table 2. The regression coefficients for the GDS-15 consistently showed a reverse association with the scores of all facets when the GDS-15 was included as one of the determinants. Nevertheless, the MBI was associated with three domains and 15 of the 26 facets of the WHOQOL-BREF before the adjustment for the GDS-15, whereas it only associated with the scores of three facets and 0 domains after the adjustment for the GDS-15.

**Table 2:** Regression coefficients and standard errors (S.E.) of depression scale measured with the GDS and the MBI for individual facets and domains of the WHOQOL-BREF under multiple linear regression models adjusted for potential confounders^†^.

	Model 1		Model 2		Model 3			
WHOQOL-BREF	MBI		GDS		MBI		GDS	
	Estimate	(S.E.)	Estimate	(S.E)	Estimate (S.E.)		Estimate (S.E.)	
Overall QOL	0.01	(0.01)	-0.06	(0.01)***	-0.00	(0.01)	-0.06	(0.01) ***
General health	0.02	(0.01) **	-0.06	(0.01)***	0.01	(0.01)	-0.05	(0.01) ***
**Physical**	0.06	(0.02) ***	-0.23	(0.04)***	0.03	(0.02)	-0.21	(0.04) ***
Pain and discomfort	0.02	(0.01)*	-0.05	(0.02)**	0.01	(0.01)	-0.04	(0.02) *
Dependence on medicinal substances and medical aids	0.01	(0.01)	-0.05	(0.02)**	0.01	(0.01)	-0.04	(0.02) *
Energy and fatigue	0.01	(0.01)*	-0.07	(0.01)***	0.01	(0.01)	-0.06	(0.01) ***
Mobility	0.03	(0.01)***	-0.08	(0.02)***	0.02	(0.01)*	-0.07	(0.02) ***
Sleep and rest	0.00	(0.01)	-0.07	(0.02)***	-0.01	(0.01)	-0.07	(0.02) ***
Activities of daily living	0.02	(0.01)**	-0.05	(0.01)***	0.01	(0.01)	-0.04	(0.01) ***
Work capacity	0.01	(0.01)**	-0.05	(0.01)***	0.01	(0.01)	-0.04	(0.01) **
**Psychological**	0.06	(0.02)***	-0.28	(0.04)***	0.02	(0.02)	-0.26	(0.04) ***
Positive feelings	0.01	(0.01)*	-0.07	(0.01)***	0.01	(0.01)	-0.06	(0.02) ***
Spirituality/ religion/personal beliefs	0.01	(0.01)	-0.07	(0.01)***	0.00	(0.01)	-0.07	(0.02) ***
Thinking/learning/memory/ concentration	0.01	(0.01)*	-0.06	(0.01)***	0.00	(0.01)	-0.05	(0.01) ***
Bodily image and appearance	0.02	(0.01)**	-0.07	(0.01)***	0.01	(0.01)	-0.07	(0.01) ***
Self-esteem	0.02	(0.01)***	-0.07	(0.01)***	0.01	(0.01)*	-0.05	(0.01) ***
Negative feelings	0.02	(0.01)**	-0.10	(0.01)***	0.01	(0.01)	-0.09	(0.02) ***
**Social relationships**	0.02	(0.02)	-0.18	(0.04)***	-0.00	(0.02)	-0.18	(0.04) ***
Personal relationships	0.01	(0.00)*	-0.05	(0.01)***	0.00	(0.01)	-0.05	(0.01) ***
Sexual activity	0.01	(0.01)	-0.06	(0.01)***	0.00	(0.01)	-0.06	(0.01) ***
Practical social support	0.00	(0.00)	-0.03	(0.01)*	-0.00	(0.01)	-0.03	(0.01) *
**Environment**	0.03	(0.01)*	-0.21	(0.03)***	0.01	(0.01)	-0.21	(0.04) ***
Freedom, physical safety and security	0.01	(0.01)	-0.09	(0.01)***	-0.01	(0.01)	-0.10	(0.01) ***
Physical environment (pollution/ noise/ traffic/ climate)	0.00	(0.01)	-0.05	(0.01)***	-0.00	(0.01)	-0.05	(0.01) **
Financial resources	0.01	(0.01)	-0.10	(0.02)***	-0.00	(0.01)	-0.10	(0.02) ***
Opportunities for acquiring new information and skills	0.02	(0.01)***	-0.05	(0.01)***	0.02	(0.01)*	-0.03	(0.01)*
Participation in and opportunities for recreation/leisure activities	0.02	(0.01)**	-0.06	(0.01)***	0.01	(0.01)	-0.05	(0.02)***
Home environment	-0.00	(0.00)	-0.03	(0.01)**	-0.01	(0.01)	-0.04	(0.01)**
Health and social care: accessibility and quality	0.00	(0.00)	-0.02	(0.01)*	0.00	(0.01)	-0.02	(0.01)*
Transport	0.01	(0.01)*	-0.03	(0.01)**	0.01	(0.01)	-0.03	(0.01)*

*p<0.05,

**p<0.01,

***p<0.001.

†Model 1 adjusted for confounders of gender, age, education, marriage, household cohabitants, MMSE, MBI, and multi-morbidity (including diabetes, hypertension, heart disease, stroke and cancer); Model 2 is adjusted for all as Model 1 plus the GDS with the exception of the MBI; Model 3 is adjusted as Model 1 plus the GDS.

GDS = Geriatric Depression Scale; MMSE = Mini-Mental State Examination; WHOQOL = World Health Organization Quality of Life Scale-Brief Version; MBI = Modified Barthel Index.

The mediation effects of depression (as assessed by the GDS-15) on the relationship between the MBI and WHOQOL-BREF score were tested, and the results are summarised in Table 3. It appears that the depression (as assessed by GDS-15) may be an MV of the relationship between the MBI and the physical, psychological, and environmental domains of the WHOQOL-BREF. However, depression (as measured by GDS-15) did not meet Baron and Kenny’s [31] criteria of a mediator of the relationship between the MBI and the social domain. The results of the Sobel test for mediation indicated that the indirect associations between the MBI and the three domains (physical, psychological, and environmental) of the WHOQOL-BREF through the GDS-15 were significant.

**Table 3:** Regression analyses testing the mediation of GDS in the relationship between MBI and four domains of WHOQOL-BREF.

	Predictor	Outcome	Estimate(S.E)	*p value*	R^2^	Sobel test statistic, *p value*
Phy Dom						4.43, <0.001
Step 1	MBI	Phy Dom	0.06(0.02)	<0.001	0.10	
Step 2	MBI	GDS	-0.14(0.02)	<0.001	0.16	
Step 3	GDS	Phy Dom	-0.23(0.04)	<0.001	0.16	
Step 4	GDS	Phy Dom	-0.21(0.04)	<0.001	0.15	
	MBI		0.03(0.02)	0.120		
Psy Dom						4.95, <0.001
Step 1	MBI	Psy Dom	0.06(0.02)	<0.001	0.07	
Step 2	MBI	GDS	-0.14(0.02)	<0.001	0.16	
Step 3	GDS	Psy Dom	-0.28(0.04)	<0.001	0.16	
Step 4	GDS	Psy Dom	-0.26(0.04)	<0.001	0.16	
	MBI		0.02(0.02)	0.125		
Soc Dom						not applicable
Step 1	MBI	Soc Dom	0.02(0.02)	0.138	0.05	
Step 2	MBI	GDS	-0.14(0.02)	<0.001	0.16	
Step 3	GDS	Soc Dom	-0.18(0.04)	<0.001	0.10	
Step 4	GDS	Soc Dom	-0.18(0.04)	<0.001	0.10	
	MBI		-0.00(0.02)	0.841		
Env Dom						4.95, <0.001
Step 1	MBI	Env Dom	0.03(0.01)	0.017	0.05	
Step 2	MBI	GDS	-0.14(0.02)	<0.001	0.16	
Step 3	GDS	Env Dom	-0.21(0.03)	<0.001	0.13	
Step 4	GDS	Env Dom	-0.21(0.04)	<0.001	0.13	
	MBI		0.01(0.01)	0.725		

Each regression is adjusted for age, gender, education, marriage, household cohabitant, MMSE, multi-morbidity (including diabetes, hypertension, heart disease, stroke and cancer).

WHOQOL = World Health Organization Quality of Life Scale-Brief Version; MMSE = Mini-Mental State Examination; MBI = Modified Barthel Index; GDS = Geriatric Depression Scale; Phy Dom = Physical domain; Psy Dom = Psychological domain; Soc Dom = Social relationship domain; Env Dom = Environmental domain

## Discussion

This study demonstrates that in community-dwelling older adults without severe physical dependence, depression (as assessed by the GDS-15) affects every facet and domain of the WHOQOL-BREF. It also implies the possibility that depression may mediate the relationship between ADL and QOL. We will examine the internal validity of this study before making further inferences.

Based on the notion that improvements in depression are associated with a better QOL in older adults with depression [4], we further explored the relationship between depressive symptoms and QOL in community-dwelling older adults. In this study, rather than selecting people with diagnosed depression, we included only relatively healthy (both healthy adults and those with mild ADL impairment) people from the community to avoid potential confounding by major comorbidity and/or physical disability. The average score of the GDS-15 was 3.5±3.2, with 22.3% of individuals suffering from depression (with a cut-off point of a GDS-15 score of 5). This rate is similar to the 21.2% rate of neurotic/major depression found in a previous study of community-dwelling older adults in an ethnic Chinese population in Taiwan [2]. Because we controlled for the demographic factors of levels of functional disability (ADL, measured by MBI) and multi-morbidity (including diabetes, hypertension, heart disease, stroke and cancer) in our model construction, these determinants did not confound the results of the final models.

The finding that depressive symptom scores are associated with every domain and facet of the WHOQOL-BREF (Table 2) corroborate a previous report of healthy adults [8]. The above findings are also consistent with previous studies of older people that used different modalities to measure QOL [33–35]. Moreover, although the WHOQOL-BREF and GDS-15 may share overlapping constructs on the psychological and physical domains, the fact that the latter is also significantly predictive of every facet of the social and environmental domains of the former cannot be explained by the overlapping construct alone. Thus, we tentatively concluded that the depression score is predictive of every item and domain scores of WHOQOL in healthy and mildly impaired (i.e., physical impairment that only mildly affects ADL) older adults. Under the WHO scheme of “Health in all policies” [36], it also implies the importance of considering depression management in the implementation of public policies for older adults due to the effects of depression on QOL.

Our study also examined how depressive symptoms modify the effect of the ability to perform daily activities on the QOL. In this study, the MBI showed a significant relationship with the physical, psychological and environmental domains of the WHOQOL-BREF before the adjustment for the depression scale, but the statistical significance disappeared when we included the GDS-15 score as a covariate (Table 2). Moreover, the initial predictive effects of the MBI on 15 of the 26 facets of the WHOQOL-BREF were significant, but the number of affected facets was reduced to three after the adjustment for the GDS-15 score (Table 2). These findings suggest that the relationship between MBI and the WHOQOL-BREF is modified by depression (as assessed by GDS-15), which is compatible with the findings of Bowling et al. [11].

The mediation effect of depression between ADL and QOL was tested using Baron and Kenny’s [31] procedure and the Sobel test [32]. Table 3 summarises the results and corroborates the possible complete mediation effects of depression (as assessed by the GDS-15) on the relationship between the MBI and the physical, psychological, and environmental domains of the WHOQOL-BREF. The R^2^ values of the regression equations were generally less than 0.16, as shown in Table 3, indicating the limited explanation by the independent variables for the variance of the dependent variable. This effect could be related to the small variation of the Likert scale and the generic nature of the WHOQOL-BREF questionnaire. However, it usually does not affect the validity of the relationship between currently existing variables in the regression models. As all facets of the WHOQOL-BREF are universally influenced by depression (as assessed by the GDS-15), it should not be due to the overlapping constructs between these two instruments. Moreover, an interventional study found that the administration of antidepressants improved QOL scores [6]. We also tentatively concluded that the mediation effect may be true and deserves further corroboration. These findings may suggest that an older adult with mildly impaired ADL may not necessarily experience a poor QOL if depression can be properly managed.

This study has the following implication for the policy regarding older adults. At the public level, depressive symptoms significantly affect every domain of the QOL, including environmental and social relationships. This finding implies that the public efforts in every aspect would be more effective if depression could be properly managed. At the personal level, because the effects of ADL on QOL may be mediated by depressive symptoms, such improvement in depressive symptoms implies a crucial way, other than restoring physical function, to improve the QOL of older adults. All of these findings suggest that depression screening in different settings and convenient access to depression treatment may improve the QOL of older adults.

The limitations of the current study are as follows. First, the magnitude of the regression coefficient appears trivial or to have limited clinical significance in the individual facets. However, because a single factor is able to predict a consistent effect on all domains and facets of the WHOQOL-BREF, this study raises an important need for further studies of geriatric depression on QOL. Second, because the participants were conveniently recruited from community centres rather than selected under randomisation, a selection bias is possible. However, because the government of Taiwan supports these community centres, they are typically located inside each community with easy and equal accessibility to older adults. The participants, therefore, may be more likely to represent the population of older adults who are physically capable in the community. This assertion is supported by the fact that the proportion of depressive individuals in this study (22.3%) is similar to that described in a previous study (21.7%, [2]). Thus, our sample would be representative of ambulatory, community-dwelling older adults who may also come from primary care settings but would be quite different from those confined to bed or repeatedly hospitalised, as our participants were able to walk for more than 100 meters without resting. Third, we did not inquire about the participants’ medical history of psychological problems and medications, which may have resulted in an underestimation of the prevalence of depressive symptoms. Similarly, the potential confounding effect of polypharmacy is not fully addressed under the current study design, although the most common chronic diseases were included in our analysis. However, depressive conditions were measured using the GDS-15 and showed the expected results. This affirms that the GDS-15 instrument is able to detect an association regardless of any special treatment for depression. Fourth, this study design is cross-sectional, which limits its power for causal inference. We recommend a prospective, longitudinal follow-up study combined with repeated measurements and a mixed effects model in the future for the purpose of corroborating the mediation effect of depressive symptoms on the relationship between ADL and QOL.

## Conclusions

Depression affects the QOL in older adults. Furthermore, depression likely plays a mediating role in the relationship between physical function impairment and QOL. In other words, an older adult with mild physical challenges may still live with good QOL if their depressive symptoms are effectively managed. We recommend taking depressive symptoms into core consideration when interpreting patient-reported outcomes and/or making policies related to older adults. In addition, depression screening in different settings and convenient access to depression treatment may improve the QOL of older adults. A cohort follow-up study is suggested to corroborate our hypothesis.
